# Acellular Wharton’s Jelly, Potentials in T-Cell Subtypes Differentiation, Activation and Proliferation

**DOI:** 10.34172/apb.2020.074

**Published:** 2020-08-09

**Authors:** Mehdi Talebi, Hojjatollah Nozad Charoudeh, Ali Akbar Movassaghpour Akbari, Behzad Baradaran, Tohid Kazemi

**Affiliations:** ^1^Department of Applied Cell Sciences, School of Advanced Medical Sciences, Tabriz University of Medical Sciences, Tabriz, Iran.; ^2^Immunology Research Center, Tabriz University of Medical Sciences, Tabriz, Iran.; ^3^Drug Applied Research Center, Tabriz University of Medical Sciences, Tabriz, Iran.; ^4^Hematology and Oncology Research Center, Tabriz University of Medical Sciences, Tabriz, Iran.

**Keywords:** Wharton's Jelly, T-cell, Immunotherapy, T-cell subsets, Differentiation, Cell orientation

## Abstract

***Purpose:*** Because of different potentials of T-cell subtypes in T-cell based cellular immunotherapy approaches such as CAR-T cell therapies; Regarding the high cost of the serum-free specific culture media, having distinct control on T-cell subset activation, expansion and differentiation seem crucial in T-cell expansion step of cell preparation methods. By the way, there was no clear data about the effect of acellular Wharton’s Jelly (AWJ) on T-cells expansion, activation or differentiation status. So, we have launched to study the effect of AWJ on T-cell’s immunobiological properties.

***Methods:*** CD3+ T-cells were isolated from healthy bone marrow allogeneic donors, sorted by FACS method and cultured on either routine phyto-hemagglutinin complemented and different concentrations of AWJ, lag phase and doubling time of the cells calculated from cell growth curve. After 3, 7 and 14-days T-cell subtypes cell markers and cell activity related genes expression rate have been evaluated by flow cytometry and real-time polymerase chain reaction (PCR) methods respectively.

***Results:*** AWJ in a 1:1 ratio compared with contemporary lymphocyte culture media showed significant activating and proliferative capacities. The introduced condition has not affected the frequency of CD4+ subpopulation of T-cells, but significantly increased even CD8+ cells and immune-activator genes in T-cells. The regulatory and memory subsets of T-cells in this study have not affected significantly.

***Conclusion:*** the study results revealed that AWJ can be utilized as a supportive substance to increase the memory properties of the T-cells, gives control to design a selective medium for expanding and differentiating memory T-cells, relatively.

## Introduction

Cellular products process engineering needs having exact knowledge about every step of the known process and having control over each of them. As well, based on world major GMP (Good Manufacturing Practice) regulatory the more minimizing the manipulating cellular products the secure the products. One of the cellular products branches immune-cell therapy, and especially T-cell based adaptive immune therapy approaches have growing interests during recent years. The CAR-T cell therapy approaches are one of the most interesting ones that utilize the antibody-based chimeric antigen receptors (CARs) on T-cells to recognition tumor-specific antigens for effectively removing hematologic and solid tumor cells.^1,2^ CAR-T cell (primarily called T body) was firstly described at Weizmann Institute of Science in Israel by Eshhar et al.^3^ T-cells naturally are composing of different subtypes that will be described below and the composition of the in an individual at different infective/inflammations and/or reactive states results in various immunological, even activating and suppressive conditions. And so, producing the CAR-T cells will utilize different subtypes of T-cells even effector or memory cells, the importance of this approach highlighted in Louis et al and Xu et al reports.^4,5^ For example, Gattinoni and colleagues reported that CAR-T-cells produced from T memory Stem cell (T_SCM_) that highly expressing CCR-7, CD95 and CD62L, shown a more durable and effective anti-tumoral effect in comparison with central memory T cells.^6,7^ Generally, CD4+ T-cell subtypes, Th1, Th2, Th9, Th17, Th22, Treg and Tfh (Follicular helper T cells), that characterizing with various surface markers and secreting cytokine profile^7^ and demonstrate different patterns of reactivity during activation process.^8^ Also, it has been reported that different T-cell subtype’s cytokine compositions demonstrated various levels and patterns of cytotoxicity,^9^ called Cytokine Storm, which can be a major limitation and regarded as important limitations of the CAR-T cell therapy approaches. In the same way, CD8+ T-cells subtypes; T_N_ (Naïve T cells), T_SCM_ (Stem cell memory T cells), T_CM_ (Central memory T cell), T_EFF_ (Effector T cells), T_EM_ (Effector memory T cells) demonstrating different immune response pattern during the or CAR-T-cells related reactivity processes. For example, Berger et al demonstrated that central memory CD8+ T-cells demonstrated long-term adoptive immune transfer compared with T_EFF_ cells in the primate model.^10^ Other researchers showed the significant effect of the optimum CD8+ and CD4+ combination of T-cells in adoptive immunotherapy experiments.^11^ By the way, it has been demonstrated that CD4+ cells support the development of CD8+ memory characterizations,^12^ these findings supporting the ideas about the importance of introducing the right combination of T-cell subsets in adoptive T-cell based immunotherapy methods.

Wharton’s jelly (WJ), is a relatively rigid connective tissue surrounding umbilical cord vessels,^13^ it contains a significant amount of mucoid extracellular matrix components which are composed of collagen, hyaluronic acid, and different sulfated proteoglycans. Reports showed that WJ is a rich source of growth factor peptides, such as insulin-like growth factor-1 and platelet-derived growth factor (PDGF), fibroblast growth factor at lower level^14^ and transforming growth factor b (TGF-b).^15^ These growth factors may accumulate within Wharton’s jelly to support the cells (e.g., MSCs). Wharton’s jelly Mesenchymal stem cells are in close interplay with their extracellular matrix. Bakhtiyar and colleagues demonstrated the secre^16^ tum of WJ-MSCs enhances wound healing in vitro.^17^ Taipale et al demonstrated the importance of them which are linked to controlling cell proliferation, differentiation, synthesis and remodeling of the extracellular matrix.^18^ The effects of acellularized Wharton’s jelly itself has not been investigated considering T lymphocytes development, proliferation or activation. Based on the Bakhtiyar’s work on the effect of AWJ on wound healing, we assumed that acellular gelatinous Wharton’s jelly (AGWJ) increases T-cell proliferation, activation and alters the subsets profile during relatively long-term culture. So, during current work, we compared the T-cells activation, proliferation, composition variations and differentiation to subtypes aiming to introduce the more natural way to produce culture mediums with the ability to prepare the best profile of T-cells utilizing in cellular immunotherapy approaches.

## Materials and Methods

### Acellular Wharton’s jelly preparation

AWJ was prepared based on previous methods described by Bakhtiyar et al.^17^ Briefly 15-20 cm sterile umbilical cords were obtained from cesarean operations, from Al-Zahra gynecology and obstetrics hospital, Tabriz, Iran. The 10 cm of cords were opened longitudinal, a vein and two arterials were removed and the jelly material scraped by sterile scalpel and approximately 5 mL of that placed in a 50 mL tube along with complete DMEM (approximately same as the jelly volume) media and resuspended by 5 mL pipette up and downing to breakdown the jelly. Then the tube content centrifuged at 200-400 g for 10 minutes. The debris and cells were pelleted and the supernatant separated and frozen at -80°C.

### T-cell isolation and culture

T-cells were separated from leukapheresis sterile samples prepared from allogeneic bone marrow donor samples referred to Shahid Ghazi hospital laboratory for CD34+ and CD3+ cells counting. The healthy eligible donors had been conditioned by subcutaneous rhG-CSF (Neopagen) 5-7 μg/kg/d 5-7 days prior to cell apheresis procedure. Around 12%-18% of the PBMCs were T-cells. Briefly, 100 μL PBMC sample collected from healthy bone marrow donors incubated with 5 μL anti-CD3-FITC (BD, USA) antibody in darkness for 45 minutes and washed by sterile PBS and resuspended in PBS solution. Then the CD3+ cells gated on lymphocytes region and approximately 0.5×10^6^-1×10^6^ cells sorted by the FACSCalibur Flow-cytometry system (BD, USA). The cells centrifuged immediately after sorting in 4°C and transferred to 10ml RPMI-1640+10% FBS with 100 IU penicillin/streptomycin medium and 5 μg/mL Phyto-Hemagglutinin (PHA) (Gipco, Germany). The cell viability evaluated by the exclusion of Trypan blue dye method and formulated as 10^4^/mL cells for next step culturing methods.

In the test groups, three different V:V (standard medium: AWJ) (1:1, 1:5, 1:10) have been evaluated. The used culture medium was the same as the control group except for the PHA, which has been minimized to around 1 μg/mL. Each medium cultured for 14 days and selected surface markers at 3^rd^, 7^th^ and 14^th^ days evaluated by flow cytometry and gene expression evaluations done by real-time polymerase chain reaction (PCR) methods.

### Cell growth curve drawing 

T-cells cultured in 12well culture plate in 10^4^/cm^2^ concentration in RPMI-1640+10% FBS +5 μg/mL PHA for first 3 days every 8hrs the cells counted by Trypan blue exclusion method (1:1 well-agitated cell medium and Trypan Blue) and counted by neobar hemocytometer. After 3 days counting continued till the 15^th^ day of culture and the medium changed every 3 days. The growth curved plotted based on the time of culturing and cell counted and growth cure plotted in a semi-logarithmic arrangement. Population doubling time (PDT) derived from each group. For calculating PDT, the estimated middle of log phase for each culturing condition cell count and the next time’s cell counts used based on routine formula as, Doubling time = [duration * log(2)\log(Final Concentration) - log(Initial Concentration)].

### Cell surface markers evaluations

At 3^rd^, 7^th^, 14^th^ days the cells harvested from each concentration group, 200-500 × 10^3^ cells prepared for CD markers analyzing. CD4-FITC, CD8-PE, CD25-FITC, FOXP3-PE and CD127-FITC based on single or panel orders. Briefly 3-5 μL of respective antibody-incubated for 5 minutes to 1 hour with cells in darkness and washed by PBS, the pellet resuspended in 500-1000 μL PBS and analyzed by BD-FACSCallibur بlow cytometry systems. For intracellular markers, permeabilization steps prior to antibody steps have been done based on manufacturer instructions.

### Immunologically important genes expression 

The second portion of the harvested cell’s (0.5 × 10^6^- 1 × 10^6^ cell) RNA extracted by Sinnagen RNX RNA extraction kit (Sinnaclone, Iran) and cDNA produced by Smobio (China) cDNA synthesis kit, based on manufactures instruction. The qPCR performed by Corbett (Rotorgene, Thermo, Germany) system. The genes and primers list mentioned in Table 1. The PCR performed by Amplicon SYBR Green qPCR master mix according to manufacturer instruction.

**Table 1:** Primers list used for important immunological response genes expression

**Gene Name**	**Primers Sequences**	
IL-2	F	ACCAGGATGCTCACATTTAAGTTTT
	R	GAGGTTTGAGTTCTTCTTCTAGACACTG
IL-10	F	GCCGTGGAGCAGGTGAAG
	R	GAAGATGTCAAACTCACTCACTCATGGCT
IFNg	F	AGCTCTGCATCGTTTTGGGTT
	R	GTTCCATTATCCGCTACATCTGAA
TGF-b1	F	CGAGAAGCGGTACCTGAAC
	R	TGAGGTATCGCCAGGAATTGT
GZMA	F	GGGACGATGTGAAACCAGGA
	R	AGGCTTCCAGCACAAACCAT
B-actin	F	GGCACCCAGCACAATGAAG
	R	GCCGATCCACACGGAGTACT

### Statistical analyzing 

The descriptive data expressed as frequency and mean ± SD, and for parametric data, an independent sample *t* test performed. For intergroup comparisons, one-way ANOVA tests have been performed, and for non-parametric or nominal parameters χ^2^ and Mann-Whitney U tests performed by GraphPad Prism v. 8.0.2. *P* value <0.05 regarded as statistically significant.

## Results

### Growth curve variations analyzing 

**Figure 1:**
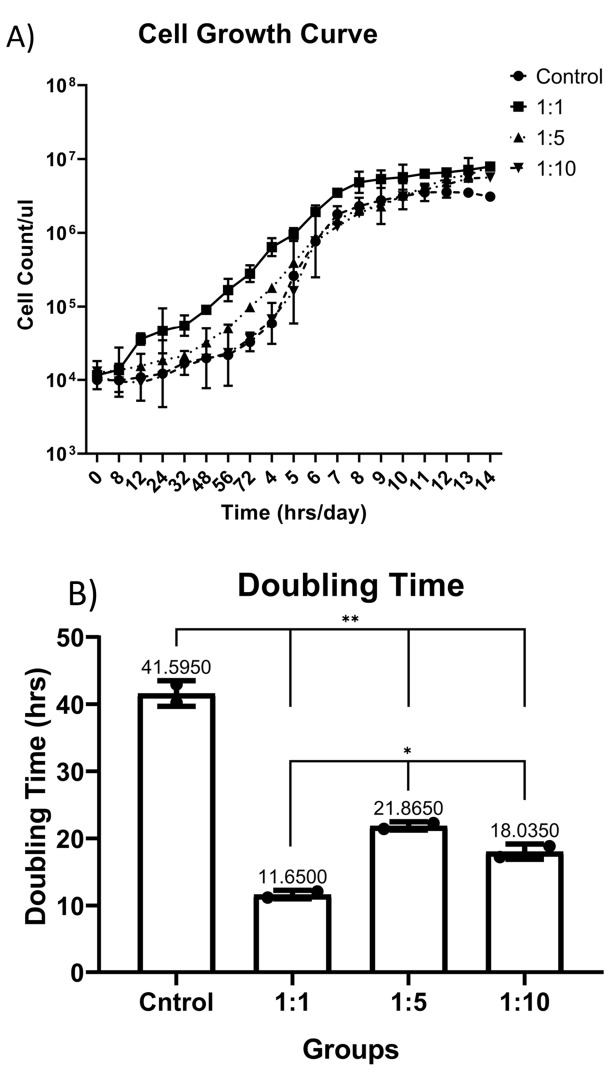
**(A)** Cell Growth Curve plotted in different concentrations of AWJ.**B)** Population doubling time (PDT) has been calculated based on the middle of the log phase time.

Growth curve plotted for control and 1:1, 1:5 and 1:10 V:V concentrations of AWJ with routine complete RPMI1640 medium, Figure1A, by the way, the PDT for control group was 42.6 ± 1.9, for 1:1 concentration group 11.65 ± 0.63, 1:5 concentration group 21.8 ± 0.61 and for 1:10 concentration group the PDT was 18.0 ± 1.13, based on the results, AWJ in 1:1 concentration rate has significant effect on proliferation (r = 0.762, *P* value = 0.023) (Figure 1).

### T-cell subtypes ratio evaluation 

**Figure 2:**
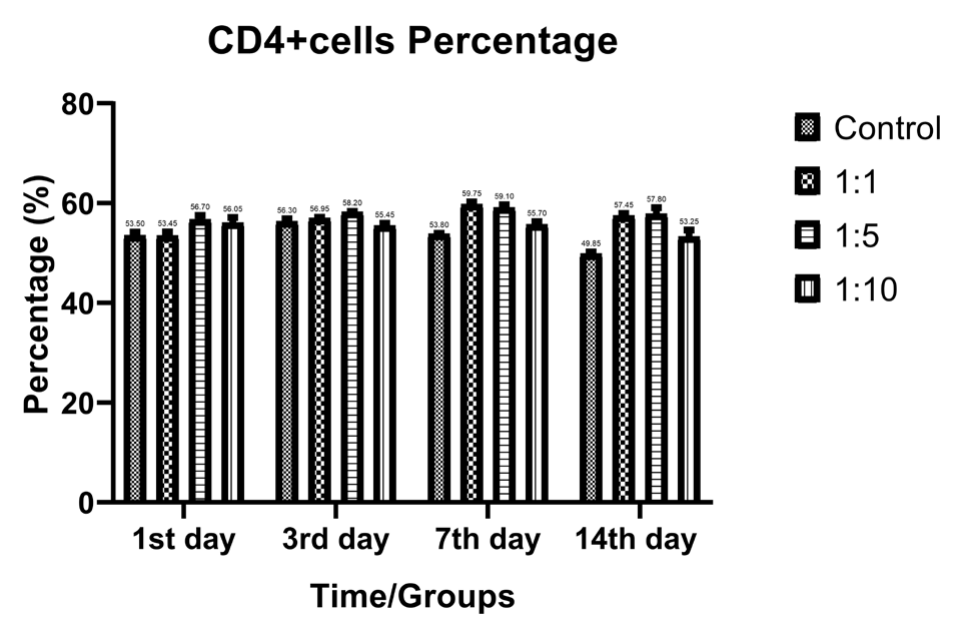
CD4+ positive subpopulation (Helper T-cells) frequency ratios of the T-cells during time in various AWJ:Culture Media. No significant variations observed between groups.

**Figure 3:**
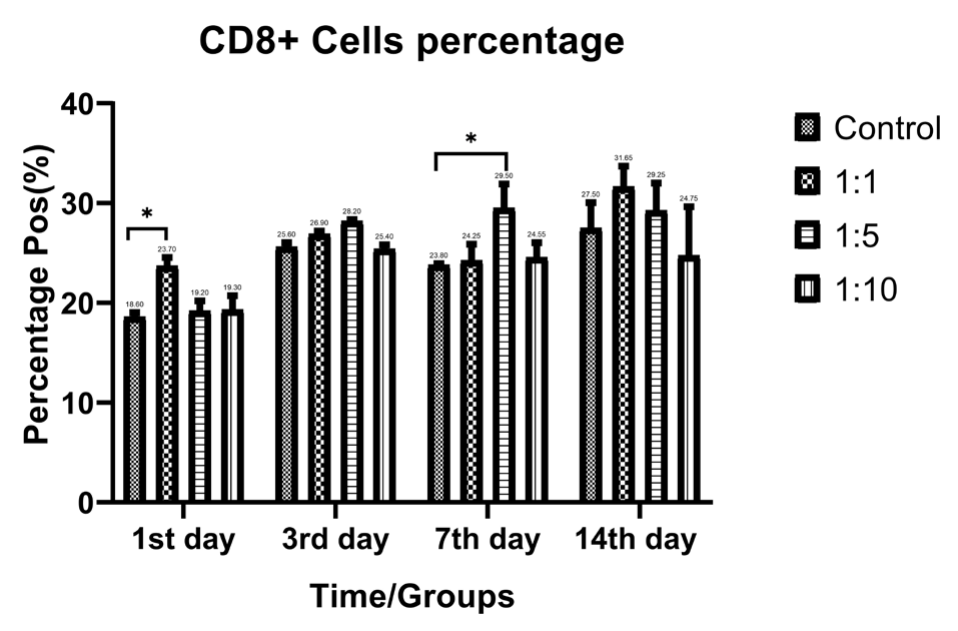
CD8+ positive subpopulation (Cytotoxic T-Cells) frequency ratios of the T-cells during time in various AWJ:Culture Media. *P value < 0.05.

**Figure 4:**
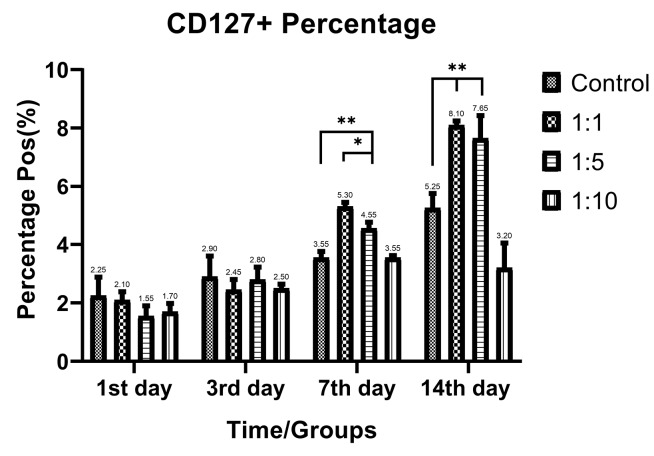
CD127+ positive subpopulation (memory T-cells) frequency ratios of the T-cells during time in various AWJ:Culture Media. *P value<0.05, **P value<0.001.

**Figure 5:**
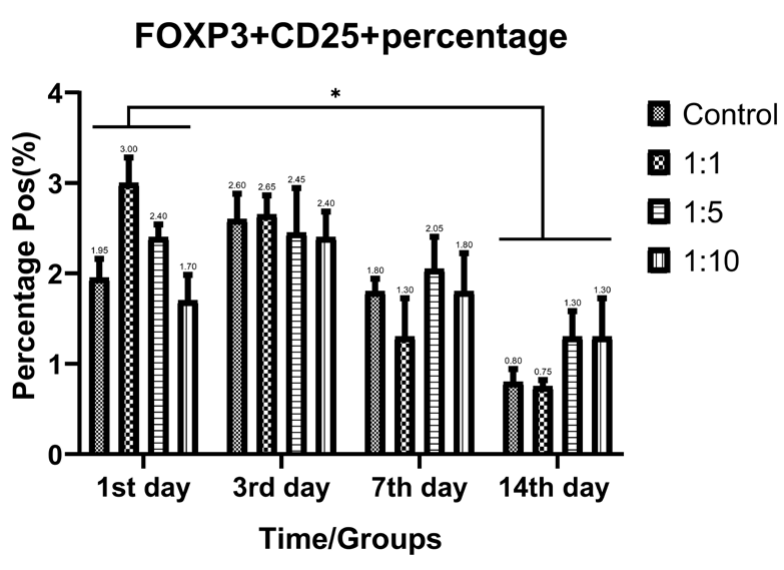
CD25+FoxP3+ positive subpopulation (regulatory T-cells) frequency ratios of the T-cells during time in various AWJ:the Culture Media. *P value<0.05.

Despite the proliferative effect of AWJ, there was no statistically significant effect on CD4+ cells. Either between concentrations (*P* value = 0.0711) and time durations (*P* value = 0.1371; Figure 2). Totally, there was no statistically significance in CD8+ cytotoxic T-cell population amount during time progression but 1:1 AWJ concentration after 24 hours and 1:5 concentration at 7^th^ day showed significant increasing (*P* value = 0.022, *P* value = 0.048, respectively) (Figure 3). About the memory T-cell subset (CD127+) population, it has been shown that there was no statistically significant relationship between different AWJ concentration groups (Figure 4), but time duration causes a statistically significant increase on memory cells concentration, may be independent of AWJ’s biologic effect (*P* value < 0.001). CD25+FoxP3+ population frequency has been evaluated as regulatory T-cell subsets in this study, and it had been shown that there was no statistically significant about the effect of AWJ on regulatory T-cell differentiation during this experiment time period, but significant decreasing after two weeks of culturing observed (Figure 5).

### Immunologically important genes expression patterns

**Figure 6:**
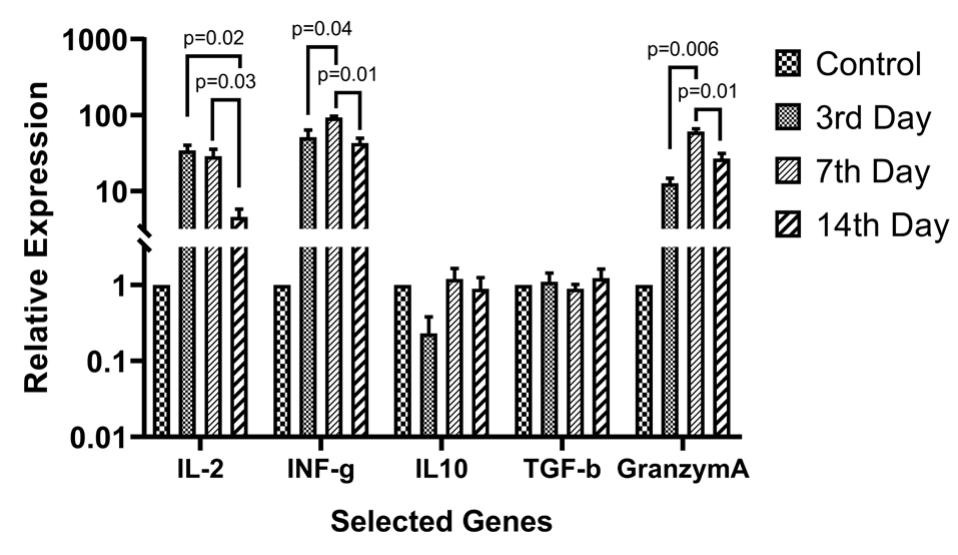
Relative expression of the selected T-cell subsets activation related genes. The 1:1 AWJ:Culture Media ratio group has been selected as the maximum activation group and IL-2, IL-10, IFN-g, TGF-b, and Granzyme A relative expression evaluated. *P* value of the statistically significant variation between groups mentioned in the picture.

Based on the growth curve data, 1:1 AWJ concentration has been chosen as the main group for evaluating the T-cell subtypes selective genes relative expressions. The expression level of each gene has been normalized with the expression levels in the control group. Generally, the expression status of the genes that related to the activation of either CD4+ and CD8+ subsets were increased as well as, regulatory and suppressive status-related genes like IL-10 and TGF-b expression were not changed significantly. Figure 6 abstracted the main finding in this part.

## Discussion

Looking after natural substances with maximum compatibility with human biology especially in rapidly growing cellular products industry seems crucial and is one of the major challenging points. Also, animal originated cellular or serum products (for example, FBS) potential dangers, also should be regarded. Regarding the high cost of serum-free specific media for different cells and also targeting the simplified methods for specific cell population selection or expansion forcing researchers to introduce new materials for fulfilling all the above-mentioned issues. Cell growth supporting properties of AGWJ have been investigated by Bakhtiyar et al they have been attributed the growth enhancement to wound healing properties to α2-macroglobulin in majority.^17^ Also, they investigated the AWJ exosomes effect on wound healing and shown the same finding.^16^ The effect of the α2-macroglobulin on lymphocytes biology has been reviewed in James work^19^ shown that the protein has effects on lymphocyte proliferation by lectins like PHA and inhibits lymphocytes activity also the results were controversial. There is no obvious research about the cell growth effect of the AWJ on hematopoietic lineages yet. As there is plenty of knowledge about great antigenicity of the amniotic fluid, that can be used as a general T-cell activator, potentially. Amniocentesis will not ethical for this purpose, so be hypothesized that the AWJ can be the surrogate to this general activator roll of amniotic fluid. Based on over result the AWJ can act as mitogens for T-cells in combination with contemporary substances like rhIL-2 or anti-CD3 (OKT3). By the way as the sudden and high dose activation of the T-cells can cause exhaustion on cells, mild activation speed may be helpful to reduce exhaustion and increase memory T-cells. Our result showed this phenomenon too.

Zelenay et al reported the progressive expression of CD25 upon activation of CD25-FoxP3+ T-cells,^20^ we evaluated the CD25+ portion of the cells alone and reducing the percentage of these cells by time may be described as this manner.

About the cell growth variation effect of AWJ, there was no study postulating the expression IGFR, PDGFR and other growth factor receptors on either activated or naïve T-cells, so the growth-supporting effect of the AWJ may be attributed to the substances other than growth factors effect, possibly lectin-like carbohydrates in AWJ milieu may serve general activating function in the manner similar to PHA.

## Conclusion

In conclusion, the current study results revealed that AWJ can be utilized as a supportive substance to increase the memory properties of the T-cells, and gives control to design a selective medium for expanding and differentiating memory T-cells, relatively.

In this study, we did not perform markers evaluation for determining central or effective memory T-cells so the additional more detailed study to evaluate the exact subtypes of either CD4+ or CD8+ T-cells are suggesting.

## Ethical Issues

All the research processes ethically certified by Iran national committee for Ethics in biomedical Researches as IR.TBZMED.REC.1397.181 and IR.TBZMED.REC.1397.611.

## Conflict of Interest

Authors declare no conflict of interest in this study.

## Acknowledgments

This study granted by Tabriz University of Medical Sciences, Research Vice-Chancellor, Faculty of Advanced Medical Sciences of Tabriz University of Medical Sciences (Reg. No: 60321) and endorsed by Ethical Committee in Medical Researches (IR.TBZMED.REC.1397.611).
